# Acute Compartment Syndrome of the Thigh After Contusion in a Football Player

**DOI:** 10.7759/cureus.53617

**Published:** 2024-02-05

**Authors:** Guilherme Correia, Pedro Mendes Santos, João Pedro Campos, Nuno Camelo Barbosa, Luís Carvalho

**Affiliations:** 1 Orthopedics and Traumatology, Hospital de Braga, Braga, PRT; 2 Orthopedics and Traumatology, Unidade Local de Saúde de Matosinhos, Matosinhos, PRT

**Keywords:** compartment syndrome, blunt trauma, anterior thigh, shoelace technique, sports trauma

## Abstract

Acute compartment syndrome of the thigh is an exceptionally uncommon condition that can have severe consequences if not promptly and effectively treated. A 19-year-old man presented to our emergency department with severe and progressive pain in his left thigh after sustaining a direct trauma during a football game 24 hours prior. Compartment pressure was assessed, confirming the diagnosis of compartment syndrome arising from a sizable intramuscular hematoma without detection of any other contributing factors. Fasciotomy incisions were closed using the shoelace technique with excellent functional results. This case highlights the importance of high suspicion and intra-compartmental pressure measurement to diagnose this condition accurately.

## Introduction

Acute compartment syndrome of the thigh (ACST) is infrequent and can lead to severe outcomes if not promptly recognized and addressed [1]. The compartments within the thigh are notably larger when compared to those in the forearm and leg. Substantial expansion of compartment volume is required to cause compartment syndrome [2]. While cellular anoxia, leading to pain that exceeds the visible extent of injury, is typically the primary indicator linked to the emergence of acute compartment syndrome (ACS), relying solely on clinical observations has been demonstrated to possess insufficient diagnostic accuracy. Sensitivity in isolation ranges from 13% to 54%. It is advisable to employ intra-compartmental pressure (ICP) monitoring for at-risk patients. This is due to the well-documented heightened sensitivity (94%) and specificity (98%) associated with diagnosing acute compartment syndrome using a slit catheter technique and a differential pressure threshold of 30 mmHg sustained over two hours [3]. Blunt trauma is the primary cause of ACST, contributing to 90% of reported cases [4], and the most affected compartment is the anterior, as it is more vulnerable to trauma [5]. ACST is an infrequent occurrence in sports, with sparse epidemiological data available. In this context, minimal consensus exists concerning the exact diagnostic criteria and the most suitable treatment methodology [6]. We present a case of ACST resulting from a contusion, which was managed surgically, along with the subsequent follow-up until the return to sports activities.

## Case presentation

**Figure 1:**
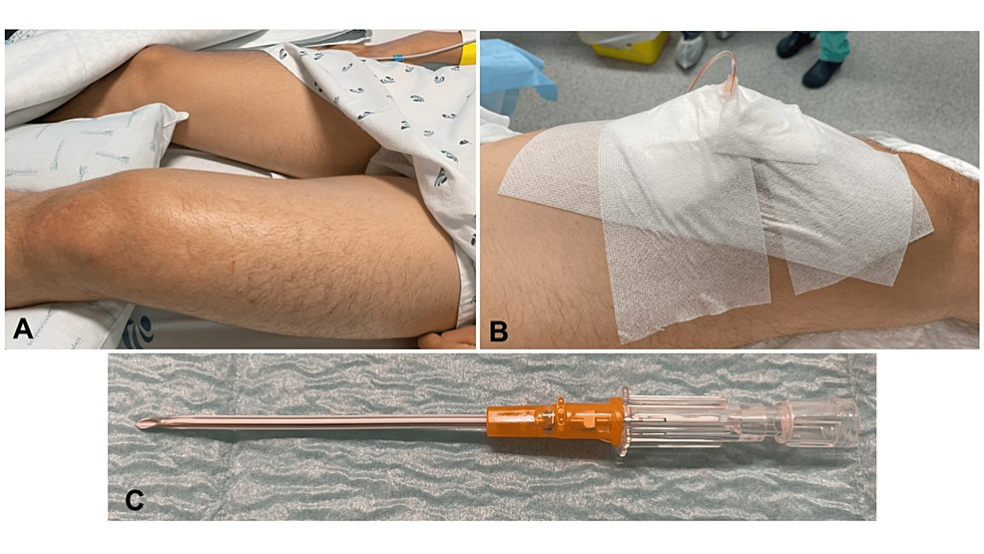
Clinical presentation A: swelling of the left thigh; B: slit catheter positioned on the anterior thigh compartment; C: slit catheter used to measure intra-compartmental pressure (ICP)

A 19-year-old male, otherwise healthy and not taking any medications, sustained a direct blow from an opposing player during a football game and developed progressive severe pain in his anterior left thigh. After the contusion, he could not continue to play, but he was able to walk home. He presented to our emergency department 24 hours after trauma and was unable to bear weight on his left lower extremity as it aggravated his thigh pain. On physical examination, he had severe swelling and paresthesia on his anterior left thigh muscle, with a circumference difference of three centimeters relative to the contralateral thigh (Figure 1). The patient had difficulty performing active knee extension. The examiner managed to achieve complete passive extension of the patient's knee but encountered limitations in flexing it beyond 30°. Plain radiographs revealed no signs of fractures. The medial and dorsal compartments were supple. His pain continued to get worse as time passed, even though he was being given narcotics. With high suspicion of ACS, he was admitted to our intermediate care unit to perform continuous ICP monitoring, according to our institution's protocol, based on work done by Duckworth and McQueen [7]. The ICP measured varied between 84-104 mmHg with corresponding delta pressure (ΔP) varying between -12 and -32, way below 30, which confirmed the ACST.

**Figure 2:**
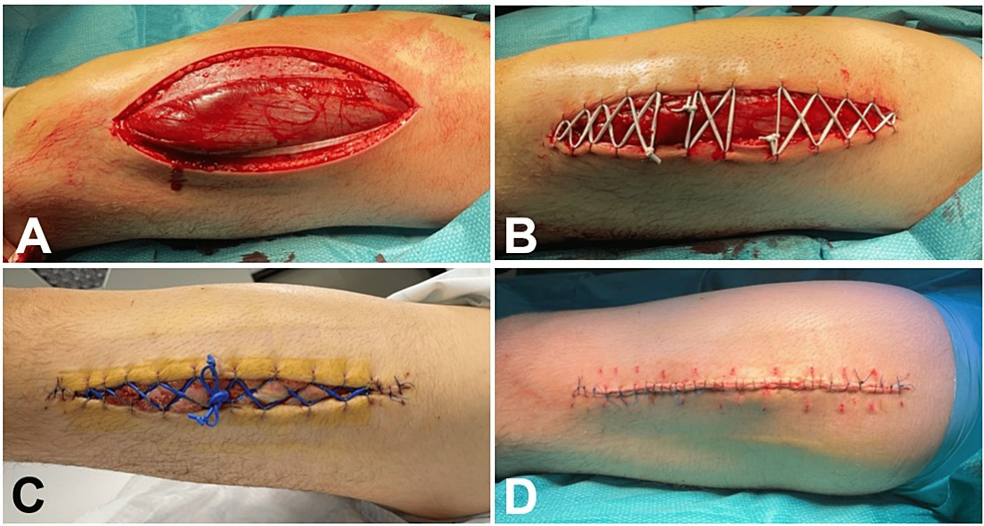
Decompressive fasciotomy A: fasciotomy of the anterior compartment through anterolateral approach; B: elastic suture with silastic vessel loop (shoelace technique); C: elastic suture on the fifth day after fasciotomy; D: secondary closure on the seventh day after fasciotomy

He was submitted to a decompressive fasciotomy of the anterior compartment through an anterolateral incision of the skin (Figure 2A) followed by placement of an elastic suture, also known as the shoelace technique (Figure 2B) with silastic vessel loops, according to the technique first described by Berman et al. [8].

**Figure 3:**
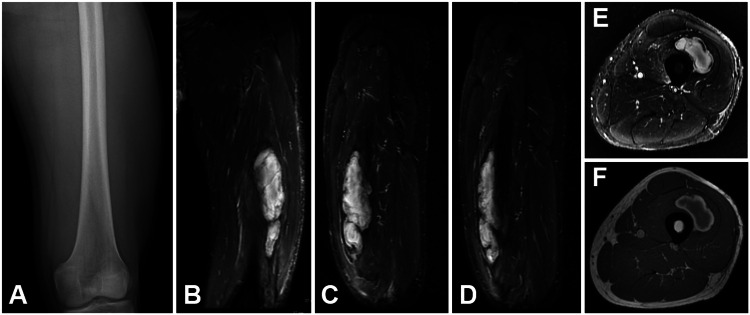
Imaging at presentation A: radiograph of the left femur at initial presentation without any signs of fracture; B: coronal MRI STIR sequence of left thigh showing large hematoma (15x3 cm); C,D: sagittal MRI STIR sequence of left thigh; E: axial MRI STIR sequence of left thigh; F: axial MRI T1 sequence of left thigh STIR - short tau inversion recovery

Afterward, magnetic resonance imaging (MRI) confirmed a large hematoma in the anterior thigh compartment adjacent to the vastus intermedius (Figure 3).

**Figure 4:**
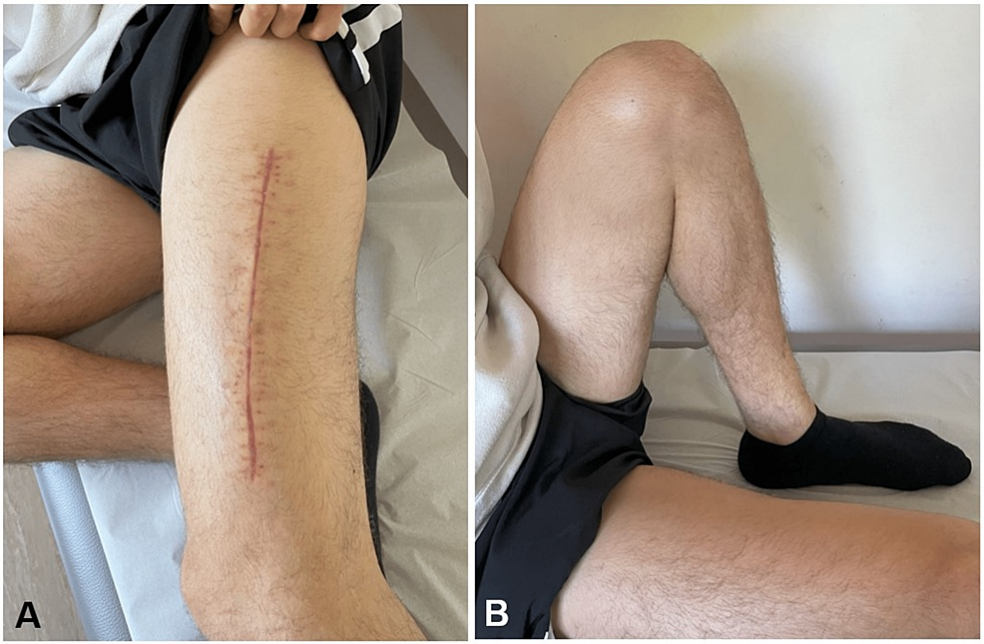
Clinical presentation at four weeks post-fasciotomy A: fasciotomy surgical wound healed; B: full flexion of the left knee

In the following days, we cleaned the wound and re-tensioned the shoelace every 48 hours. On the seventh day after admission, the surgical wound had no signs of tension or infection, so we closed the fascia, subcutaneous tissue, and skin. On the first day post-closure, he started assisted passive range of motion (ROM) of the knee, with a flexion set-point of 45º, progressing to 90º on the second day after surgery. On the third day, the patient was discharged with an indication to continue to mobilize the knee without weight bearing. In the fourth postoperative week, he recuperated his full ROM, 0-145º (Figure 4), and started weight bearing and a quadriceps muscle strengthening program in a physiotherapy clinic. 

**Figure 5:**
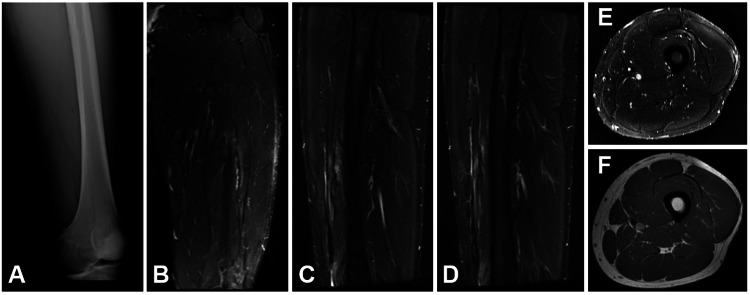
Follow-up imaging A: radiograph  of left femur at three months post-fasciotomy with no signs of myositis ossificans; B: coronal MRI STIR sequence of left thigh showing reabsorption of hematoma; C, D: sagittal MRI STIR sequence of left thigh; E: axial MRI STIR sequence of left thigh; F: axial MRI T1 sequence of left thigh STIR - short tau inversion recovery

Radiographs of the left thigh performed at six weeks and three months showed no signs of myositis ossificans. At three months, he was squatting 145 kg on a squat machine and started running drills. An MRI performed at this time point revealed complete spontaneous hematoma resorption (Figure 5). At four months, he returned to play.

## Discussion

Thigh muscle contusions are very common in contact sports, and the vast majority heal without any consequences with conservative treatment. But rarely, depending on the severity of the contusion and the formation of hematoma, pressure builds up to a point where pain does not respond even to narcotics. Within the sports context, distinguishing ordinary contusions from ACST holds crucial importance. The severity of complications depends on the duration preceding fasciotomy [9]. In our case, fasciotomy had a dramatic pain-relieving effect, and the patient regained normal ROM four weeks after surgery.

The ICP values in this case exceed the lower limit recommended for fasciotomy by Mubarak et al. and Matsen et al. (30 and 40 mmHg, respectively) [10,11], as well for the ΔP value of <30 mmHg proposed by McQueen et al. [12], thus fasciotomy was indicated. The method of ICP monitoring used in this case provided reliable results while allowing continuous monitoring and being less costly when compared to other available commercial systems like Stryker® STIC Device (Kalamazoo, MI, US). Irrespective of the measurement system employed, the patient should be supine, ensuring that the affected area aligns with the heart's level. Adequate systemic analgesia should also be provided [1].

Sports like football, rugby, American football, and ice hockey could be linked to a heightened risk of ACST [6]. In exceptional circumstances, similar to our case, athletes can develop a hematoma at the injury site due to blunt trauma, which might evolve into acute compartment syndrome [1]. In this context, an ACST could manifest without any fractures and might develop gradually, creating a significant diagnostic challenge that could lead to severe consequences resulting from tissue necrosis, fibrosis, and muscle contractures.

Fasciotomy involves making an incision in the enclosing skin and fascia of the compartment to alleviate pressure, enabling proper tissue perfusion within the compartment. Consequently, due to the swelling of edematous muscles, the skin edges of the fasciotomy wound retract, and even after the compartment edema subsides, the skin adheres to the underlying muscle, making the typical closure within three to five days unfeasible. Hence, skin grafting frequently becomes necessary. In this instance, the employment of elastic sutures with silastic vessel loops enabled a gradual bringing together of the skin edges over a span of seven days. This approach achieved delayed primary closure without requiring skin grafting for the widely open fasciotomy wound. This strategy effectively avoided associated drawbacks such as pain at the donor site, infection risk, incomplete graft adherence, and an unsatisfactory cosmetic outcome [8]. This technique also encompasses the use of more expensive vacuum-assisted closure (VAC) devices (mean daily cost of 135€ for VAC and 14€ for shoelace) [13]. 

As recommended by Riede et al., routine radiographs were conducted during the follow-up at six weeks and three months post-injury to rule out the possibility of myositis ossificans [14].

## Conclusions

Contusions are frequent in contact sports. Although benign most of the time, some can be complicated with acute compartment syndrome. This case highlights the importance of combining a concerning examination with intra-compartmental pressure measurement to accurately and timely identify these lesions that could be life-threatening and have long-term deleterious effects on athletes' quality of life and sporting ability.
